# Interest of FDG-PET in the Management of Mantle Cell Lymphoma

**DOI:** 10.3389/fmed.2019.00070

**Published:** 2019-04-09

**Authors:** Clément Bailly, Thomas Carlier, Cyrille Touzeau, Nicolas Arlicot, Françoise Kraeber-Bodéré, Steven Le Gouill, Caroline Bodet-Milin

**Affiliations:** ^1^CRCINA, INSERM, CNRS, Université d'Angers, Université de Nantes, Nantes, France; ^2^Department of Nuclear Medicine, CHU de Nantes, Nantes, France; ^3^Department of Hematology, CHU de Nantes, Nantes, France; ^4^Department of Nuclear Medicine, ICO-René Gauducheau, Saint-Herblain, France

**Keywords:** mantle cell lymphoma, FDG-PET, staging, therapeutic evaluation, SUV

## Abstract

FDG-PET changed response assessment and therapy strategy in diffuse large B-cell lymphoma and Hodgkin disease lymphoma. The value of FDG-PET evaluation in MCL has not been extensively studied and a recent expert consensus highlighted the need for more studies addressing this question. Data of the literature show the value of FDG-PET at baseline in patients with MCL, underlining the good sensitivity of this examination for the initial staging of this pathology, but also the potential impact of semi-quantitative analysis in this indication. The determination of SUVmax at diagnosis might indeed provide important prognostic information. Some studies also suggest the potential value of early and end-of-treatment metabolic assessment in MCL, but these results need to be validated in standardized prospective studies. These results also underlie the need to integrate FDG-PET results into MCL treatment strategy to improve disease management in identifying patients who might benefit from more intensive therapy.

## Introduction

The use of positron emission tomography with 18Fluoro-deoxyglucose (FDG-PET) in patients with malignant lymphomas has increased dramatically in the last decade, both for initial staging and for therapeutic evaluation. FDG-PET has indeed become an essential tool in the management of these patients, and regular meetings of international expert committees, such as those held annually in Menton or Lugano, have allowed standardization of practices (1–3). Currently, the role of FDG-PET in rarer histological subtypes of lymphomas such as mantle cell lymphoma (MCL) is less well-defined. MCL is an aggressive subtype of non-Hodgkin lymphoma (NHL) which accounts for ~5% of all NHLs (4, 5). The majority of patients presents with advanced-stage disease and often has extra-nodal sites of involvement such as the gastrointestinal (GI) tract and bone marrow. Patients diagnosed with this disorder generally have poor prognosis and even if the introduction of novel approaches combining rituximab and chemotherapy increased the median overall survival (OS), most patients still experience relapse (6–8). However, a small minority of patients seems to have a longer survival than would be expected and achieve long lasting remissions. The lack of early biomarkers has become a major issue in MCL. Recent advances in the understanding of the clinical, molecular, and genetic characteristics of MCL have identified prognostic factors that might be useful to develop risk-adapted therapies (6, 8). These prognostic factors include, inter alia, splenomegaly, performance status, mitotic index, and the Mantle Cell International Prognostic Index (MIPI) (9). The role of FDG-PET and its prognostic value in MCL remain debatable as contradictory results regarding its utility for assessing disease burden and response to therapy. In this era of personalized medicine, a non-invasive method for assessing tumor heterogeneity and predicting survival or response to therapy could permit a better selection of worse prognosis patients who might benefit from more intensive therapy. A recent expert consensus highlighted the need for more studies addressing this question (2).

## Methodology of the Literature Review

The literature search entailed a systematic search of MEDLINE and PUBMED for publications that were published between 2000 and 2018 using the following key words: mantle cell lymphoma, PET, FDG, SUV.

### FDG-Pet at Diagnosis In MCL

FDG-PET is recommended by international guidelines for initial staging in all FDG-avid histological subtypes of lymphomas including MCL (2, 3).

The first correlation between FDG uptake and the histopathological subtype of lymphoma as defined by the WHO classification was published in 2003 (10). This retrospective study showed that FDG-PET detected at least one pathological site in all cases of MCL Indeed, existing data in the literature confirm this observation and show that FDG-PET at diagnosis in patients with MCL has a high sensitivity in the detection of lesions in nodes and spleen (11–14). However, the sensitivity for bone marrow and gastrointestinal (GI) involvement is inadequate to replace routine bone marrow and GI biopsy in disease staging (11).

Besides, despite this significant uptake of FDG in all patients with MCL, most of the literature show a significant intra-individual and inter-individual heterogeneity with uptake. Indeed, the values of SUVmax, the metric widely adopted as a surrogate of the overall net rate of FDG uptake, varied between 2.5 and 36.7 and between 1.0 and 18.8 in the series of Mato et al. (15) and Bodet-Milin et al. (11), respectively. In this latter, a calculated intra-patient SUVmax gradient was ≥5 in 46% of cases and ≥10 in 13%. Thus, intra- and inter-individual differences might reflect heterogeneity in tumor cell biology, especially since the study by Schöder et al. (16) reported that the value of SUVmax is potentially correlated with histological aggressiveness. Oncogenesis of MCL being a multistep process (17), progressing from a less to a more aggressive form, it can therefore be postulated that low SUVmax value is related to less aggressive MCL cells while high SUVmax values reflects a more aggressive behavior or a more advanced disease. Existence of intra-individual SUVmax variation in MCL might be similar to what is observed in Richter's syndrome where aggressive transformation is located to a specific tumor tissue area. The results of the retrospective study published by Karam et al. confirmed this findings, showing an adverse impact on both event-free-survival (PFS) and overall survival (OS) for MCL patients presenting with SUVmax >4.8 (18). This is also supported by the results of the LyMa-PET study (19). In this prospective ancillary study of the multicentric LyMa trial (NCT00921414), studying the predictive value of FDG-PET at diagnosis in young previously untreated MCL patients, high SUVmax (>10.3) was associated with shorter PFS (*p* = 0.0003) and OS (*p* = 0.0003). This observation at diagnosis was not found in works by Schaffel et al. (ASH 2009), Mato et al. (15) and Bodet-Milin et al. (11) even if this latter reported a negative trend of an SUV max> 6 on the overall survival (*p* = 0.07). In addition, this close relation between tumor cell biology and SUVmax in MCL is also supported by the relationship between SUV max, blastoid variant considered as the most aggressive form of MCL and high percentage of Ki67 positive MCL cells (19). SUVmax measurement could therefore be used to assess tumor cell aggressiveness as FDG-PET has the advantage to be a whole-body non-invasive technique, not restricted as Ki-67 immunostaining to tissue biopsies. The prognostic value of SUVmax seems even reinforced when associated with clinical and biological factors, as shown by Bodet-Milin et al. (11). Used together, IPI and SUVmax allowed to separate MCL patients into three groups with different PFS duration: low (29%; no relapse/progression), intermediate (42%; median PFS: 37 months) and high risk (29%, median PFS: 22 months) (*p* = 0.004) (11). Preliminary results of the LyMa-PET study (19) seem to confirm this observation. This approach clearly identified a subset of patients with a very high risk of early progression after first line treatment, who might benefit from more intensive therapy.

### FDG-Pet for Response Assessment In MCL

#### Response Assessment During Treatment

The most recent international recommendations for the use of imaging in malignant lymphoma does not mandate FDG-PET-based response assessment in MCL outside the context of a clinical trial (2). These conclusions based on a limited number of publications are due to the lack of prospective data, the heterogeneity of patient populations/treatment strategies, and most importantly, the lack of uniformity in the way 18FDG-PET imaging is obtained and interpreted. Thus, although some studies showed no significant predictive value for interim FDG-PET in terms of PFS or OS (14, 15, 20, 21), some reported higher progression rate for patients exhibiting a positive interim FDG-PET, irrespective of therapy applied and particularly before autologous stem-cell transplant (ASCT) (22–25). The preliminary results of the Lyma-PET study seem to confirm these data by demonstrating the potential prognostic value of the variation of SUVmax called ΔSUVmax (defined as the percentage of reduction of SUVmax between PET at baseline and PET before ASCT) on OS and PFS (19).

Interestingly, in the Nordic MCL3 study (22) and the work by Htet et al. (25), the authors described inferior PFS and OS predicted by FDG-PET positivity before-ASCT and detectable minimal residual disease (MRD) after transplant. Because these techniques were reported as having independent prognostic values, both may be of importance to guide treatment decisions. Yet, in the Czech Lymphoma Study Group-MCL1 observational study (26), only achievement of FDG-PET–negativity independently correlated with PFS. In this prospective analysis, the safety and efficacy of alternation of R-CHOP and R-cytarabine for elderly/comorbid MCL patients ineligible for high dose therapy or ASCT was explored, with initiation of Rituximab maintenance (RM) in most of them. In this group, a survival benefit of RM was observed for patients achieving response by FDG-PET response criteria regardless of their MRD-status (26, 27).

If all of these data suggest the potential value of early metabolic assessment in MCL patients, prospective studies are warranted to validate these results.

#### Response Assessment After Treatment

FDG-PET is the standard of care for remission assessment in FDG-avid lymphoma, yet its value in MCL is debatable. In their retrospective series of 44 patients, Bodet-Milin et al had shown significantly lower PFS for patients with residual FDG-uptake at the end of treatment, according to the IHP criteria (11). Mato et al. confirmed this prognostic value of negative FDG-PET at the end of treatment, again using the IHP criteria, in 53 patients with MCL, with better PFS at 3 years and a trend for OS (*p* = 0.07) (15). Similar results have also been reported by Brepoels et al who demonstrated better PFS at 2 years for patients who achieved a complete response at end-of-treatment FDG-PET (57 vs. 22%, *p* = 0.011) (20). However, some studies have also shown contradictory results such as Kedmi et al. (21) and Hosein et al. (14), who found no significant difference on survival between MCL patients with positive or negative FDG-PET findings at the end of treatment.

Thus, even if these different results seem to show a certain prognostic impact of FDG-PET at the end of treatment, published data are currently too heterogeneous to allow definitive conclusions to be drawn. In addition, some of these publications suffer from many methodological biases. Particularly, none of these latter used the Deauville Score, as actually validated and recommended by Lugano's Recommendations in Lymphoma (2), except for Lamonica et al. (28) yet in patients with relapsed or refractory MCL. Recent guidelines enacted to standardize PET protocols and to ensure more reproducible analyses between scans and centers will hopefully soon lead to the full integration of these interpretation criteria into future prospective investigations.

## Conclusion

FDG-PET changed response assessment and therapy strategy in diffuse large B-cell lymphoma and Hodgkin disease lymphoma. The value of FDG-PET evaluation in MCL has not been extensively studied and a recent expert consensus highlighted the need for more studies addressing this question. Data of the literature show the value of FDG-PET at baseline in patients with MCL, underlining the good sensitivity of this examination for the initial staging of this pathology, but also the potential impact of semi-quantitative analysis in this indication. The determination of SUVmax at diagnosis might indeed provide important prognostic information. Some studies also suggest the potential value of early and end-of-treatment metabolic assessment in MCL, but these results need to be validated in standardized prospective studies. These results also underlie the need to integrate FDG-PET results into MCL treatment strategy to improve disease management in identifying patients who might benefit from more intensive therapy.

## Author Contributions

All authors listed have made a substantial, direct and intellectual contribution to the work, and approved it for publication.

### Conflict of Interest Statement

The authors declare that the research was conducted in the absence of any commercial or financial relationships that could be construed as a potential conflict of interest.
